# Reliable Discrimination of 10 Ungulate Species Using High Resolution Melting Analysis of Faecal DNA

**DOI:** 10.1371/journal.pone.0092043

**Published:** 2014-03-17

**Authors:** Ana Ramón-Laca, Dianne Gleeson, Ivor Yockney, Michael Perry, Graham Nugent, David M. Forsyth

**Affiliations:** 1 EcoGene®, Landcare Research, Auckland, New Zealand; 2 Institute for Applied Ecology, University of Canberra, Canberra, Australia; 3 Landcare Research, Lincoln, Canterbury, New Zealand; 4 Landcare Research, Palmerston North, Manawatu, New Zealand; 5 Arthur Rylah Institute for Environmental Research, Department of Environment and Primary Industries, Heidelberg, Victoria, Australia; Smithsonian Conservation Biology Institute, United States of America

## Abstract

Identifying species occupying an area is essential for many ecological and conservation studies. Faecal DNA is a potentially powerful method for identifying cryptic mammalian species. In New Zealand, 10 species of ungulate (Order: Artiodactyla) have established wild populations and are managed as pests because of their impacts on native ecosystems. However, identifying the ungulate species present within a management area based on pellet morphology is unreliable. We present a method that enables reliable identification of 10 ungulate species (red deer, sika deer, rusa deer, fallow deer, sambar deer, white-tailed deer, Himalayan tahr, Alpine chamois, feral sheep, and feral goat) from swabs of faecal pellets. A high resolution melting (HRM) assay, targeting a fragment of the 12S rRNA gene, was developed. Species-specific primers were designed and combined in a multiplex PCR resulting in fragments of different length and therefore different melting behaviour for each species. The method was developed using tissue from each of the 10 species, and was validated in blind trials. Our protocol enabled species to be determined for 94% of faecal pellet swabs collected during routine monitoring by the New Zealand Department of Conservation. Our HRM method enables high-throughput and cost-effective species identification from low DNA template samples, and could readily be adapted to discriminate other mammalian species from faecal DNA.

## Introduction

Reliably identifying the species occupying an area is fundamental to many ecological and conservation studies, but identifying even large mammals may be difficult if the species is at low density and/or lives in densely forested habitats [1], [2]. Many mammals, and especially large herbivores, deposit numerous faecal pellets (scats) daily [3]. Unfortunately, identifying species using faecal morphology is unreliable, particularly if multiple species with similar pellets are potentially present in the study area [4]–[6]. Recent advances in the collection, preservation and amplification of mammalian faecal DNA (e.g. [7], [8]) mean that genetic methods can now be used to identify mammal species from pellets [9], [10]. However, only a few studies have developed a protocol for using faecal DNA to discriminate among multiple mammalian species [11], [12].

New Zealand has no native land mammals but many species of ungulate (Order: Artiodactyla) were deliberately introduced into New Zealand during 1851−1926 [13] and 12 species are considered extant in the North, South and Stewart Islands [14], [15]. Following establishment, most ungulate populations increased to high densities [16] that had substantial negative impacts on native ecosystems [17]–[19]. Over many decades, considerable resources have been expended reducing the abundances of ungulates in order to minimise their adverse impacts [20], [21] and prevent range expansions [14]. There is an ongoing need for better tools for detecting ungulates so that their distributions and abundances can be monitored. However, detecting secretive and wary ungulates in closed-canopy forest can be difficult [1], [3] and there are many places in New Zealand where multiple species of wild ungulate are present [14]. Thus, although faecal pellet counts have been used as an index of ungulate abundance in New Zealand forest habitats since the 1950s [22], [23], interpreting these data is difficult because many of the ungulate species present cannot be reliably differentiated by the shape, size and colour of their faecal pellets [15]. The aim of this study was to overcome that difficulty by providing a new DNA-based method that enabled faecal pellets to be reliably assigned to the ungulate species that produced them.

Faecal material is a typically inadequately preserved source of DNA because it is exposed to the environmental conditions that degrade DNA [24], [9]. In addition, faecal DNA normally includes other sources of DNA from the diet or the intestinal flora that prevail over the host DNA or endogenous DNA from the gut epithelial sloughed cells [8]. A faecal DNA-based method for identifying the ungulate species in New Zealand (Table 1) needs to be reliable, have the ability to start from low DNA template, be applicable to high-throughput protocols, and be cost-effective. A species-informative gene with little intraspecific variability is needed to identify species accurately. To overcome the problems of low template and degradation, successful DNA amplification can be enhanced by targeting a short fragment of a mitochondrial gene because mitochondrial DNA is found in greater copy numbers in a cell and a shorter DNA fragment amplification will predominate in the PCR reaction [25]. Most current ungulate molecular identification studies from faeces make use of the PCR amplification and sequencing approach [4], [5], [26], [6]. A higher-throughput and more cost-effective approach is needed if large numbers of samples are to be processed. High Resolution amplicon Melting (HRM) Polymerase Chain Reaction (PCR) analysis using a fluorescent dye [27] was identified as the procedure most likely to satisfy our requirements. Importantly, HMR-PCR is a closed-tube assay (meaning less cross-contamination than other methods), that does not require post-PCR actions because the necessary reagents are all included in the same step [28]. The principle behind HRM analysis is that differences in length and nucleotide composition will result in different melting temperatures [29], [30]. Species discrimination relies on unique ‘disassociation’ or melting temperature curve patterns that will enable species identification when compared to control samples [31], [32]. HRM-PCR begins with standard quantitative amplification in the presence of an intercalating saturating dye that fluoresces when DNA is double-stranded, providing information about target DNA quantity. The amplification step is followed by a gradual DNA denaturation that releases the intercalating compound as the DNA becomes single-stranded and thus loses its fluorescent property [29]. Changes in fluorescence are recorded by a camera, enabling direct and instant analysis from the HRM-PCR computer without having to undertake any post-PCR steps (e.g. gel electrophoresis, PCR product quantification and purification, Sanger sequencing, capillary electrophoresis). HRM analysis is used widely in human medicine [33] but has seldom been used for vertebrate species identification (but see Morgan *et al*. [32] and Sakaridis *et al*. [34]). This study features the development and use of a mitochondrial gene region to reliably discriminate 10 ungulate species through use of HRM-PCR assay of faecal DNA. The feasibility of using the method in the field is also addressed.

**Table 1:** Intraspecific variability observed in the Cytochrome *c* oxidase subunit 1 (COI), Cytochrome *b* (CYTB), and 12S-rRNA (12S) genes in the 10 ungulate species included in this study.

Common name	Species	COI		CYTB		12S			
		Haplotypes seen	Samples sequenced (populations)	Haplotypes seen	Samples sequenced (populations)	Haplotypes seen	Samples sequenced (populations)		
Feral goat	*Capra hircus*	2 (1 bp)	10 (2)	2 (1 bp)	10 (2)	2 (1 bp)	10 (2)		
Red deer	*Cervus elaphus*	2 (1 bp)	13 (4)	2 (1 bp)	13 (4)	1	13 (4)		
Sika deer	Cervus nippon*	1	9 (3)	1	10 (3)	1	10 (3)		
Rusa deer	*Cervus unicolor*	1	5 (2)	1	5 (2)	1	12 (2)		
Fallow deer	*Dama dama*	1	13 (1)	1	7 (1)	1	13 (1)		
Sambar deer	*Cervus timorensis*	1	8 (1)	1	8 (1)	1	8 (1)		
White-tailed deer	*Odocoileus virginianus*	-	-	1	9 (1)	1	10 (1)		
Himalayan tahr	*Rupicapra rupicapra*	2 (2 bp)	10 (1)	1	10 (1)	1		10 (1)	
Alpine chamois	*Hermitragus jemlahicus*	2 (5 bp)	12 (1)	2 (8 bp)	12 (1)	2 (5 bp)		12 (1)	
Feral sheep	*Ovis aries*	2 (2 bp)	6 (1)	2 (1 bp)	9 (1)	2 (2 bp)		9 (1)	

Intraspecific variability observed in COI (678 bp), CYTB (405 bp), and 12S (674 bp) within the 10 ungulate species and populations tested as indicated by the number of haplotypes present and the base pairs [bp] differences between each haplotype. *Red deer-like haplotype from hybrids is not considered. Feral pigs (*Sus scrofa*) and feral cattle (*Bos taurus*) were not included in this study because their scats are easily distinguished from these 10 species [15].

## Materials and Methods

### Ethics statement

All tissue and faecal samples used in this study were obtained from whole carcasses provided to us by recreational and commercial hunters from land managed by the New Zealand Department of Conservation. No animal was killed for the purposes of this study. All animals were harvested in accordance with normal recreational and commercial hunting practices as permitted by the New Zealand Department of Conservation, Wellington, New Zealand. Under New Zealand law, Institutional Animal Ethics Committee approval was not required for this study because all samples were collected from animals harvested for management purposes.

### Sampling DNA from known individuals

**Figure 1:**
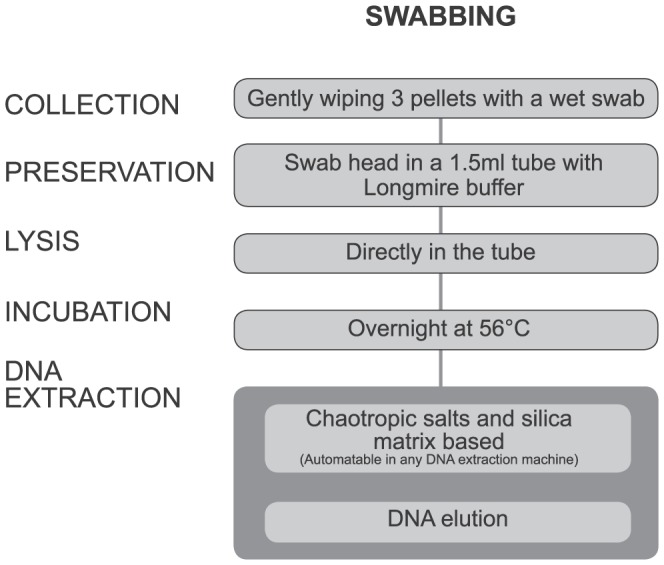
Faecal DNA preservation and extraction methods used for 10 ungulate species in New Zealand.

Fresh faeces and tissue samples were obtained from wild populations of 10 ungulate species in January–June 2012 (Table 1). Differentiation of red deer (*Cervus elaphus scoticus*) and wapiti (*Cervus elaphus nelsoni*) was not attempted, with the *Cervus elaphus* samples collected outside the wapiti area defined in Nugent [35]. At least eight individuals (including a minimum of four males and four females) of each of the 10 species were shot. Three faecal pellets were removed from the rectum of the shot animal within 60 minutes of death and swabbed with a COPAN plastic applicator sterile rayon swab dipped in Longmire lysis buffer [36] (Fig. 1). The swabs were then stored, out of direct light, in vials containing Longmire buffer. To minimise the risk of contamination with human DNA, sterile disposable gloves were used whenever faecal pellets were handled. Samples were allocated a unique identification number and labelled with the date, location and species. A sample of tissue was taken from each animal's ear for two purposes: so that results from the mitochondrial DNA sequencing could be used as reference sequences; and for use as positive controls in the HRM. The faecal and tissue samples from individual ungulates were divided into two groups: one group of individuals was used in the laboratory for assay development, assessment and reliability/repeatability checking, the other group was kept for subsequent blind testing. Sample digestion was performed directly in the sample tubes (Fig. 1) by adding 500 μl of DXT tissue digest buffer (Qiagen) and 5 μl of proteinase DX (Qiagen) followed by overnight incubation at 56°C. DNA extractions were conducted in an automated extraction machine (QIAxtractor, Qiagen) following manufacturer's instructions. DNA was then eluted in 70 or 100 μl of DXE (Qiagen) for faecal and tissue samples, respectively.

### Mitochondrial DNA analysis

Three mitochondrial DNA genes were evaluated to find the marker that was most variable between species but with minimal variation within species: cytochrome *c* oxidase subunit 1 (COI), a highly conserved region of the cytochrome *b* (CYTB), and 12S-rRNA (12S). All tissue samples were sequenced for the three genes (primers used in Tables 2 and S1) and aligned using Geneious version R6 (Biomatters: www.geneious.com). Resulting consensus sequences were used as references to create a database for future species identity assignments using the custom blast tool within Geneious.

**Table 2:** Primers used in this study.

Name	Gene	Sequence	Fragment size in HRM (bp)	[primer] μM	Reference
LCO1490	COI	GGTCAACAAATCATAAAGATATTGG			[65]
HCO2198	COI	TAAACTTCAGGGTGACCAAAAAATCA			[65]
COIelaphusF	COI	AACCGCTGATTATTTTCAACC			This study†
COIelaphusR	COI	GTAGAATAAGGATATATACTTC			This study†
Universal-50b	CYTB	GACYAATGATATGAAAAACCAYYGTTGT			[66]*
CB-N-10920	CYTB	CCCTCAGAATGATATTTGTCCTCA			[67]
12S-FWmod	12S	GGTAAATCTCGTGCCAGCC*		0.2	[51]
12S-REV	12S	TCCAGTATGCTTACCTTGTTACGAC			[51]
12S-ariesR	12S	CAGTTTAATTAAAATTTAACTCTATTTAGTATGA	112	0.25	This study
12S-damaR	12S	GGTCCTAGCTATCGTGTTTCAGCGGC	172	0.15	This study
12S-elaphusR	12S	CTTTATTTAGTATAGTGCTTTAACACA	93	0.25	This study
12S-hircusR	12S	TACTCTGGCGAATAATTTTGTTTCTG	264	0.2	This study
12S-jemlahicusR	12S	GCTTTTTACAGTTTAATTAGAATTTAACC	120	0.2	This study
12S-nipponR	12S	CTGAAGATGGCGGTATATAGACTGTAG	399	0.125	This study
12S-rupicapraR	12S	TATAGTTATGGCTTTTTACAGTTTAAC	130	0.2	This study
12S-timorensisR	12S	AGTGGGGTATCTAATCCCAGTTTGA	210	0.05	This study
12S-unicolorR	12S	GCTTAATTGGAGTTTAACTTTATTTGAA	110	0.25	This study
12S-virginianusR	12S	GTTTAACTTTATTTGGCGTAGTGCTTA	99	0.2	This study

Fragment size in the HRM in combination with primer 12-FWmod, in base pairs (bp); [primer]: final concentration in the HRM-PCR; *Modified from [68]; †Designed based on sequence Accession AB245427 from [69].

### Identifying species from faecal DNA using HRM analysis

A conserved region of the 12S-rRNA gene, from base 295 to 692 of the *Cervus elaphus* mitochondrial genome, was chosen for the identification assay because of its low intraspecific variation (Table 1) and low mutational rate [37], [38]. Species-specific reverse primers for each of the 10 species were designed so that resulting PCR products were all of different length, ranging from 93 to 399 bp, in combination with forward primer 12S-FWmod (Tables 2 and S1). Specificity of the primers was attained by positioning them at variable sites of the alignment and exploiting the 3′ end SNP of the primer sequences. Melting behaviour of the fragments was predicted using uMeltbatch^SM^ v2.0 [39]. HRM-PCR reactions were performed in a Rotor-Gene 6000 cycler with a final volume of 10 μl, containing 5 μl of 1 × Type-it HRM PCR mix (Qiagen), 1 μl of 12S-FWmod and species-specific primer mix in the concentration shown in Table 2, and 1 μl of DNA template. Cycling conditions consisted of an initial denaturation step of 5 min at 95 °C, followed by 45 cycles of 10 s at 95 °C, 30 s at 60 °C and 10 s at 72 °C. The final melting step ramped from 70 to 90 °C, with 0.1 °C increments and 2 s at each temperature. DNA at 1 ng/μl of each of the 10 ungulate species was dispensed in all runs twice for technical replicates as positive controls. Large differences in concentration can affect the resolution of HRM assays [28], [40]. Preliminary testing (data not shown) indicated that a concentration of 1 ng/μl minimised variation in the resolution of our HRM assays and hence this concentration was used for our positive controls. Raw data from the HRM-PCR were analysed using Rotor-Gene ScreenClust software (Qiagen) [41], which clusters the melting curves with the positive controls using a principal component analysis (PCA) with 3 dimensions. Results from the HRM-PCR were also analysed using the Rotor-Gene Q series software v 2.2.3 genotyping tool (HRM genotyping) that assigned species automatically based on the positive controls. Only samples with at least 50% confidence were considered, with samples below that threshold automatically indicated as variation. Melt curves difference graphs for each species were also generated. Normalization of the raw melting curves to scale the fluorescence of the samples on both software tools was set at the beginning and the end of the melting at 72–73 and 86–87°C, respectively. All discrepancies between the two identification results from the two programmes were verified by examining the difference graph of the normalised melt curve in the Rotor-Gene Q series software. Samples from four known individuals of each of the ten species were tested to assess the reproducibility and accuracy of the assay. Reliability was tested using samples from at least four individuals of each of the ten species in a blind trial. All tests were performed in duplicates as technical replicates.

### Sampling faecal DNA from unknown individuals: a field test

The feasibility of the method developed using known samples was assessed using samples of unknown origin collected in the field. New Zealand Department of Conservation (DOC) staff and contractors conducting routine faecal pellet counts [42], [43] were provided with sampling kits and instructed how to swab freshly deposited faecal pellets. The DOC is the primary government agency that monitors and manages the conservation impacts of wild ungulates in New Zealand. All field sampling was conducted during the 2012/13 austral summer. The 95 faecal DNA samples collected by DOC staff were analysed as above, except that 10% of all HRM amplified products were subjected to dye-terminator sequencing using primer 12S-FWmod to check the accuracy of the HRM analysis. Incongruent results from the PCA analysis and the difference melt graph were also subjected to dye-terminator sequencing. PCR products of samples with a threshold cycle value (C_q_) [44] (i.e. the cycle in which they reach the detection threshold) >30 were also sequenced. Sequencing products were run in a 3130xl Genetic Analyser and identified using the Geneious custom blast tool with the 12S-rRNA tissue reference sequences as reference database.

## Results

### Mitochondrial reference sequences

All 10 ungulate species were sequenced (Table 1), except white-tailed deer for COI, and species consensus sequences were aligned for each of the three genes (Files S1−3). The COI gene alignment length of nine species was 678 bp, of which 481 (70.5%) were identical between species (GenBank Accessions: KF317902-KF317915). The alignment length of the CYTB gene was 405 bp, from which 287 (70.9%) were identical (GenBank Accessions: KF317916- KF317929); the 12S alignment was 674 bp long with 527 identical sites (78.2%) (Genbank Accessions: KF317930- KF317938).

In all genes sequenced, four out of the 10 sika deer individuals showed identical nucleotide composition to red deer sequences. The other six individuals were all the same haplotype, with one close to *C. nippon yesoensis* and *C. nippon centralis* (from the main Japanese islands of Hokkaido and Honshu) according to the CYTB phylogenetic tree (built using PhyML for Geneious Neighbour-Joining TN93) and Kuwayama and Ozawa [45].

### HRM analysis of known samples

**Figure 2:**
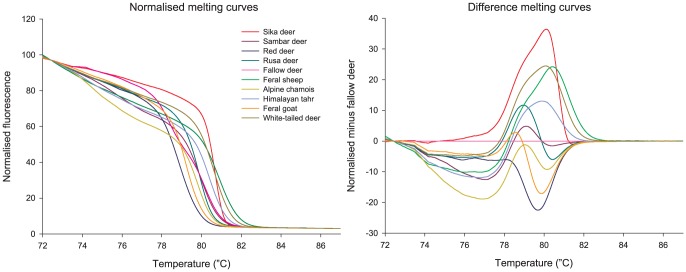
High resolution melting analysis of a fragment of 12S rRNA. Curves of positive controls of all 10 ungulate species in a normalised melting curve graph (left) and an example of a difference graph of the normalised melting curves (right) minus fallow deer. Each curve is the mean of the technical replicates of that species included in the positive controls.

All species were distinguishable by their 12S species-specific fragment melting behaviour (Fig. 2). Ungulate mitochondrial DNA was obtained from 100% of the known-species samples, with only 2.2% of miscalled replicates in the PCA and 1.1% in the HRM genotyping analysis that were readily corrected by eye when checking the difference plot in the normalised melt graph generated in the Rotor-Gene Q series software. All samples were then correctly identified with an average confidence in the HRM genotyping analysis of 85.62%. Despite the observed divergence in the genotyping confidence for some species (Table S2), there was no obvious intraspecific variation in the melting curves. The two feral goat haplotypes found within the 12S HRM-PCR target region were correctly identified.

Ungulate DNA was also obtained for 100% of the blind trial samples. Of the 42 blind samples, 39 (92.86%) were initially accurately identified and the other three showed discrepancies in one of their technical replicates between the HRM genotyping results and the PCA (3.61% of the total number of replicates) that were also corrected by checking the difference plot. Consequently, all blind trial samples were identified as their predicted species with an average of 82.8% confidence in the HRM genotyping analysis (Table S2).

### HRM analysis of unknown samples

Ungulate mitochondrial DNA was obtained from 89 (93.68%) of the 95 unknown field samples, from which 66 were identified as *Capra hircus* (feral goat), 17 as *Cervus elaphus* (red deer), and 6 as *Rupicapra rupicapra* (Alpine chamois). Of these, only one technical replicate (0.56%) with a C_q_ <30 in the PCA disagreed with the species assignment of the HRM genotyping (Table S2). This discrepancy was also corrected by looking at the difference plot in the normalised melt graph. The failure rate increased to 1.68% if samples with a C_q_ >30 were also considered. Discrepancies were also detected on the HRM genotyping analysis: 1.68% of the technical replicates with C_q_ <30 and 3.91% of the technical replicates with a C_q_ >30. Most of these failures resulted from *Capra hircus* (feral goat) samples that were misidentified as *Cervus elaphus* (red deer). Late amplification occurrence in the HRM-PCR for these samples suggested low host DNA presence. Resulting sequences from 10% of the HRM-PCR products were consistent with the results obtained from the PCA and the melt difference graph, with the exception of one *Capra hircus* sample that was misidentified as *Cervus elaphus* (the C_q_ value for both replicates of this sample was 32).

## Discussion

The method reported here reliably identifies 10 ungulate species using a multiplex HRM analysis of mitochondrial DNA extracted from swabs of faecal pellets. Managers often wish to control overabundant populations of native and non-native ungulates because of their considerable environmental and economic impacts (e.g. reviews in [46], [47]). Knowing the ungulate species present at particular locations will enable managers to better monitor changes in the distributions and abundances of ungulates in response to management [48], [49].

A highly instraspecifically conserved fragment of the 12S rRNA gene (ranging from 93 up to 399 base pairs long, depending on the species) was used for the ungulate species identification HRM analysis assay because small fragments are easier to amplify from typically degraded samples such as those extracted from faeces [25]. The 12S gene has a low mutational rate [37], [38] and has previously been used for ungulate identification [38], [50], [51]. Differentiation of species was achieved by using a species-specific primer that amplified dissimilar length fragments. The conserved-within-species region chosen is a reliable marker for ungulate species identification. Only two haplotypes, with just one variable site, were found for feral goat. Additionally, partial sequences of the cytochrome *b*, the subunit 1 of the cytochrome *c* oxidase, and the 12S subunit of the ribosome RNA of New Zealand specimens of the 10 ungulate species (except the COI sequence of white-tailed deer) are now available and new primers have been described.

Successful identification of faecal samples depends on the quality and quantity of DNA present in the sample, which will vary with the time since the pellet was deposited and environmental factors such as humidity [9]. We recommend that only faecal pellets of fresh appearance (i.e. moist, soft and usually a black or dark green colour) are swabbed in order to maximise the probability of correct species identification. While a number of often time-consuming PCR-based experiments have been developed to identify, discriminate, and detect game species (review in [52]), no HRM-based method has been described to date. Previous ‘gel-free’ mammalian species identification assays based on the T_m_ of a DNA fragment used a qPCR approach (e.g. [53]), but to our knowledge HRM has not been used to identify wild mammalian species.

The diagnostic HRM-PCR analysis used here has six key advantages over other methods. First, a 2-hour run analysis in a closed-tube performance enables high-throughput screening (approximately three times faster compared to Sanger sequencing in our laboratory) while hindering cross-contamination. Second, it is cheaper than a standard PCR and Sanger sequencing procedure (three times cheaper in our laboratory). Third, it provides an estimate of the target DNA relative concentration when compared with the positive controls, which is important for faecal DNA samples because they also contain DNA from other sources. The combined HRM-qPCR is therefore a quantitative and qualitative experiment in one [28]. Fourth, although some manual checking is desirable, species identification is automatically performed by the software, avoiding any potential bias in interpretation. Fifth, because the HRM-PCR is a non-destructive procedure, gel separation and sequencing can still be performed if desired. Sixth, because the approach requires only standard oligonucleotide primers, any laboratory with an HRM-capable qPCR machine can undertake this assay. Laboratories that cannot afford this equipment could still use the ungulate primers multiplex and identify the species by gel separation of the amplicons followed by Sanger sequencing if necessary.

Variation in template concentration resulted in differences in the ScreenClust scoring. Further examination of the normalised plot and difference plots should be undertaken to detect these abnormalities. We recommend that samples above a C_q_ of 30 cycles or below 50% confidence in the HRM genotyping are either not considered or are subjected to further identification analysis. In the latter scenario, samples should be treated very carefully and the PCR product should be sequenced to accurately assign the species. As with Vossen *et al.* [28] and Granados-Cifuentes and Rodriguez-Lanetty [40], our experiment showed that different concentrations of DNA can affect the melting behaviour and therefore the species discrimination. Although a sensitivity limitation of the approach, this potential problem can be overcome by performing a standard sequencing reaction after the HRM run. We recommend that positive controls in the HRM are 1 ng/μl and their C_q_ around cycle 25, and samples should have a similar C_q_ for optimum performance. Despite the automatic species identification when introducing the control samples in the HRM associated software, some analytical interpretation of the results, especially of the normalised difference melt plots, should be undertaken to discriminate accurately between species. All samples in the HRM should be in a similar concentration and treated in a similar way to minimise ionic differences that could affect their melting behaviour [30]. Optimisation of the PCR conditions is recommended if a different HRM-PCR mix is used since the optimal annealing temperature of the multiplex could differ between enzymes and Mg^2+^ concentrations [28].

Red-deer-like haplotypes found for the three genes examined in deer identified morphologically as sika deer suggest that sika-red deer hybridization is occurring in New Zealand. Although hybridization between sika and red deer was thought to be rare both in New Zealand [54] and in their naturally overlapping distribution due to their size difference [55], it has been found to occur commonly where one of the species has been introduced [56]-[59]. Further molecular experiments with nuclear markers, such as those used by [59], would be necessary to understand the extent of hybridization between sika deer and red deer in New Zealand. No hybrids will be detected using this species identification approach [32], whereas all the hybrids will be identified by their mitochondrial matching (i.e. their maternal line). Red-sika hybrids will therefore be identified either as red deer or sika deer using our species identification procedure.

Our analysis of faecal DNA samples collected by Department of Conservation staff and contractors during the 2012/13 austral summer confirmed that the method reported here can be used to identify ungulate species in typical New Zealand field conditions, with >90% of the swabs collected yielding a species identification. Furthermore, the costs of the faecal DNA sampling kits (NZD ∼1.5) and laboratory analyses (NZD ∼21.0) are considered low relative to the value of the information (E.F. Wright, Department of Conservation, pers. comm.). Hence, the Department of Conservation has decided to deploy the faecal DNA sampling method reported here as part of its national biodiversity monitoring system [60].

The method developed here could also assist with fraudulent meat detection [61], food safety enforcement [62], and forensic wildlife investigations [63]. The short length of the targeted gene makes it a suitable marker for degraded and poorly preserved samples that have a very low DNA copy number, and hence it may be useful in other contexts such as identifying species from saliva in browsed twigs [64].

### Conclusion

Ten closely related ungulate species can now be identified from faecal DNA using a HRM analysis of their maternal lineage. Our results also contribute to the mitochondrial study of ungulates by providing new primers and reference sequences now available in public databases. The method reported here could readily be adapted to discriminate other mammalian species from faecal DNA.

## Supporting Information

Table S1 Primer list.
(XLS)

Table S2 HRM results summary.
(XLSX)

File S1 Ungulate 12S alignment.
(NEX)

File S2 Ungulate COI alignment.
(NEX)

File S3 Ungulate CYTB alignment.
(NEX)
